# Yucca fern shaped CuO nanowires on Cu foam for remitting capacity fading of Li-ion battery anodes

**DOI:** 10.1038/s41598-018-24963-2

**Published:** 2018-04-25

**Authors:** Zhifeng Wang, Yanshan Zhang, Hanqing Xiong, Chunling Qin, Weimin Zhao, Xizheng Liu

**Affiliations:** 1 Key Laboratory for New Type of Functional Materials in Hebei Province, School of Materials Science and Engineering, Hebei University of Technology, Tianjin, 300130 China; 2 Tianjin Key Laboratory of Advanced Functional Porous Materials, Institute for New Energy Materials and Low-Carbon Technology, School of Materials Science and Engineering, Tianjin University of Technology, Tianjin, 300384 China; 3 School of Materials Science and Engineering, Central South University, Changsha, 410083 China; 4 Key Laboratory of Advanced Energy Materials Chemistry (Ministry of Education), Nankai University, Tianjin, 300071 China

## Abstract

To remit capacity fading of lithium ion battery (LIB) anodes, freestanding yucca fern shaped CuO nanowires (NWs) on Cu foams are fabricated as anodes by combining facile and scalable anodization of copper foams followed by calcination. The porous and radial configuration of the hierarchical CuO NWs on the Cu foam substrate guarantees the remarkably improved electrochemical performance with durable cycle stability and excellent rate capability compared with CuO NWs on Cu foils. The reversible capacity remains 461.5 mAh/g after 100 repeated cycles at a current density of 100 mA/g, and a capacity of 150.6 mAh/g even at a high rate of 1000 mA/g. By examining the surface morphology of the cycled samples, possible performance fading route is proposed. The 3D CuO NWs network with a porous architecture simutaneously reduces the ion diffusion distances, promotes the electrolyte permeation and electronic conductivity. This novel strategy might open a new window to develop durable CuO based composite anodes for LIBs.

## Introduction

The explosive growth of electronics market and electric vehicle industries raise stringent requirement for more reliable, cost-effective and high capacity energy storage devices. Lithium ion battery (LIB) is one of the dominant choices due to its advantages such as high energy density, high operation potential and long cycle life since their commercialization^1,2^. However, the rapid market growth requires better batteries in terms of capacity, durability and cost, which depends on the key electrode materials. The currently used graphite anode prohibited a higher specific capacity and energy density due to its relatively lower theoretical capacity of 372 mAh/g^3,4^. Therefore, it is highly urgent to develop new anode materials with higher electrochemical performance and lower cost. Transition metal oxides (M_*x*_O_*y*_ where M can be Mn, Fe, Co, Ni, Cu and so on) exhibit super Li ions storage capability (600~1300 mAh/g) through a conversion reaction mechanism, which make them promising candidates for next generation LIBs anodes^5–7^. Unfortunately, the conversion reaction processes encountered with huge volume variation, pulverization and disconnected from the current collector during discharge/charge cycles. Thus, metal oxide anodes have not yet been practical utilized. To conquer these issues, tremendous efforts are devoted to fabricating nanoscale materials such as nanoparticles, nanowires, nanorods, nanotubes, nanosheets and their electrochemical performance have been improved to some extent^8^. Another effective strategy is to integrating the transition metal oxides into conductive network such as carbon nanotubes, graphene and so on^9–11^. Most of the nanoscale metal oxides or corresponding composite are fabricated into electrode by blending with organic binders. However, the weak bonding power between commonly used binders and anode materials are usually failed to meet the electrode volume expansions. In addition, the insulation essence of organic binder deteriorates the electrochemical performance. Therefore, freestanding and binder-free novel structures and materials are in demand and being developed^9–12^.

Among various transition metal oxides, CuO is drawing great focus due to its merits such as high theoretical capacity of 674 mAh/g^13^, low cost, environmental benignity and ease of production. Nevertheless, similar with other transition metal oxides, it is still hindered by the morphological collapse caused by the volume fluctuation along with the lithiation/delithiation^13–16^. Although the conductive Cu appeared as one of the important lithiation products, it is hardly connected together to form an electrons transport way to the current collector, causing a low conductivity for the electrode composite. Facing these challenges, it is necessary to adopt new architecture design and material preparation processes. The morphology controlled copper oxides (porous microspheres, flower-like, and thorn-like CuO) were thoroughly investigated as anodes for LIBs. It is found that the CuO microspheres exhibit a superior cycle stability of 429.0 mAh/g after 50 cycles^17^. Furthermore, design and fabrication of freestanding and binder-free electrode composite could improve the conductivity and accommodation ability of volume expansions, which have been proved to be an effective strategy to enhance the electrochemical performance. Yuan *et al*. prepared CuO nanoarrays by utilizing the ammonia solution to engrave the Cu foil as free standing anode^18^. Tan *et al*. fabricated freestanding cable-like CuO/carbon-nitride core-shell nanostructures which obviously improved energy/power densities of the whole electrode^12^. Chen *et al*. developed *in-situ* etched and oxidated the specified Cu foil method for preparation the 3D network architecture with flower-like nanosheets connected by nanowires, which exhibited better electrochemical performance as anode for rechargeable batteries^19^. We recognize that freestanding materials with porous structure offer great benefits to enhance the electrochemical properties. Thus, porous nanowires combined the 3D conductive network will have superior performance as anodes for LIBs.

Herein, we report on an elaborate design of freestanding yucca fern shaped CuO nanowires (NWs)@Cu foam with hierarchical porous structure through facile and scalable anodization method and their application as anodes for LIBs. For comparison, CuO NWs@Cu foil was also prepared by the same method. Owing to the unique yucca fern shaped CuO cluster and a synergistic effect with the 3D conductive network, this CuO NWs@Cu foam composite demonstrates a better electrochemical performance compared to CuO NWs@Cu foil anode. The detailed mechanisms have been carefully examined by investigating the morphology evolution on the cycled composite electrodes. Due to the unique properties and facile preparation method, the CuO NWs@Cu foam composite displays promising applications as next-generation anode for LIBs.

## Results

**Figure 1:**
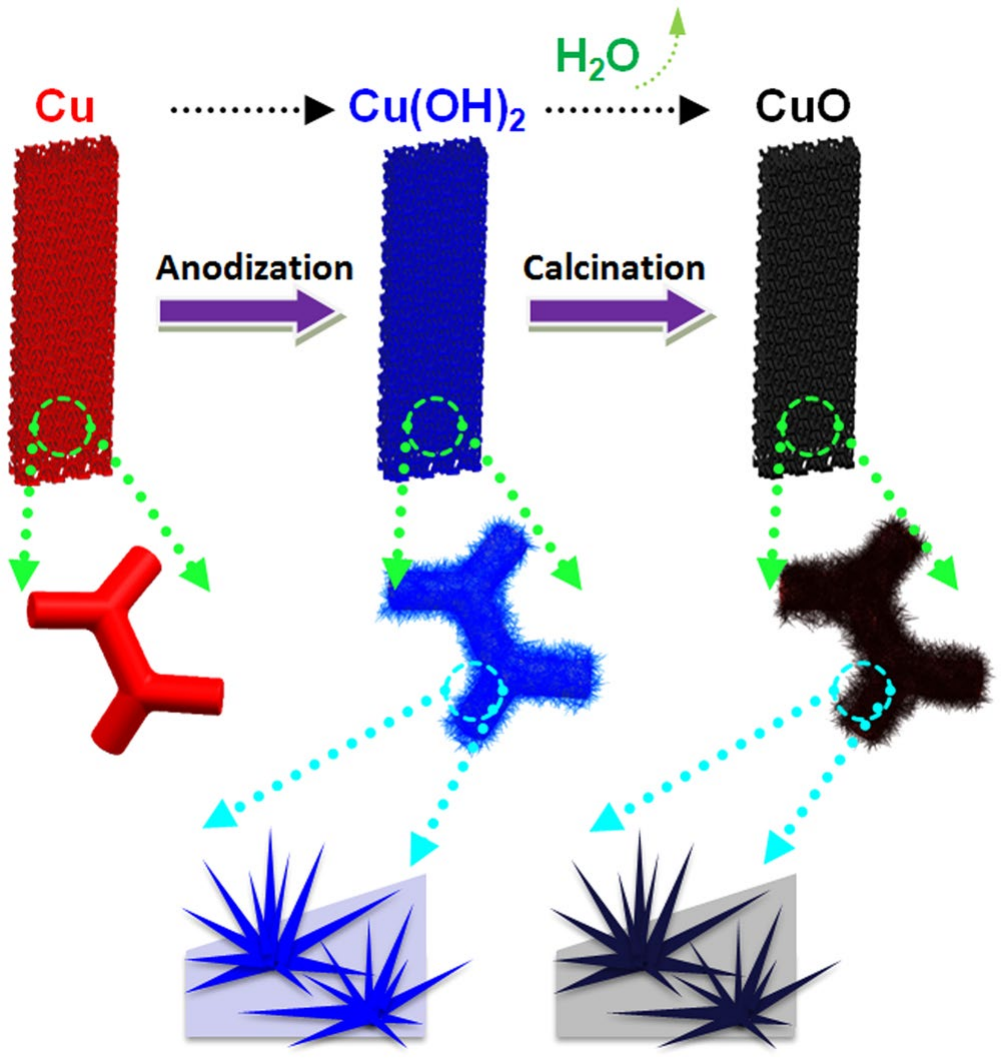
Schematic illustration of the fabrication process of hierarchical porous CuO composites.

The typical preparation process of the 3D hierarchical porous CuO NWs on Cu foam is displayed in Fig. 1. The Cu(OH)_2_ NWs clusters were first obtained through the electrochemical anodization of Cu foam in an alkaline aqueous solution. The Cu atoms at the surface were oxidized to Cu^2+^ ions and immediately reacted with OH^−^ from the alkaline solution to form Cu(OH)_2_ and deposited on the surface. The thickness and morphology can be tuned by the optimizing electrolyte concentration, current density, reaction temperature and time^20,21^. During calcination, the dehydration reaction of Cu(OH)_2_ NWs occurs at high temperature. Plenty of vacancies and pores were generated at dehydration sites of Cu(OH)_2_. As a result, CuO NWs with porous structure are finally obtained. The digital photographs of the bare Cu foam, anodized foam and heat treated sample are shown in Fig. S1. The red copper foam changed to navy blue after anodization and then to brownish black after calcination, which are corresponding to the formation of Cu(OH)_2_ and CuO on the Cu foam substrates.

**Figure 2:**
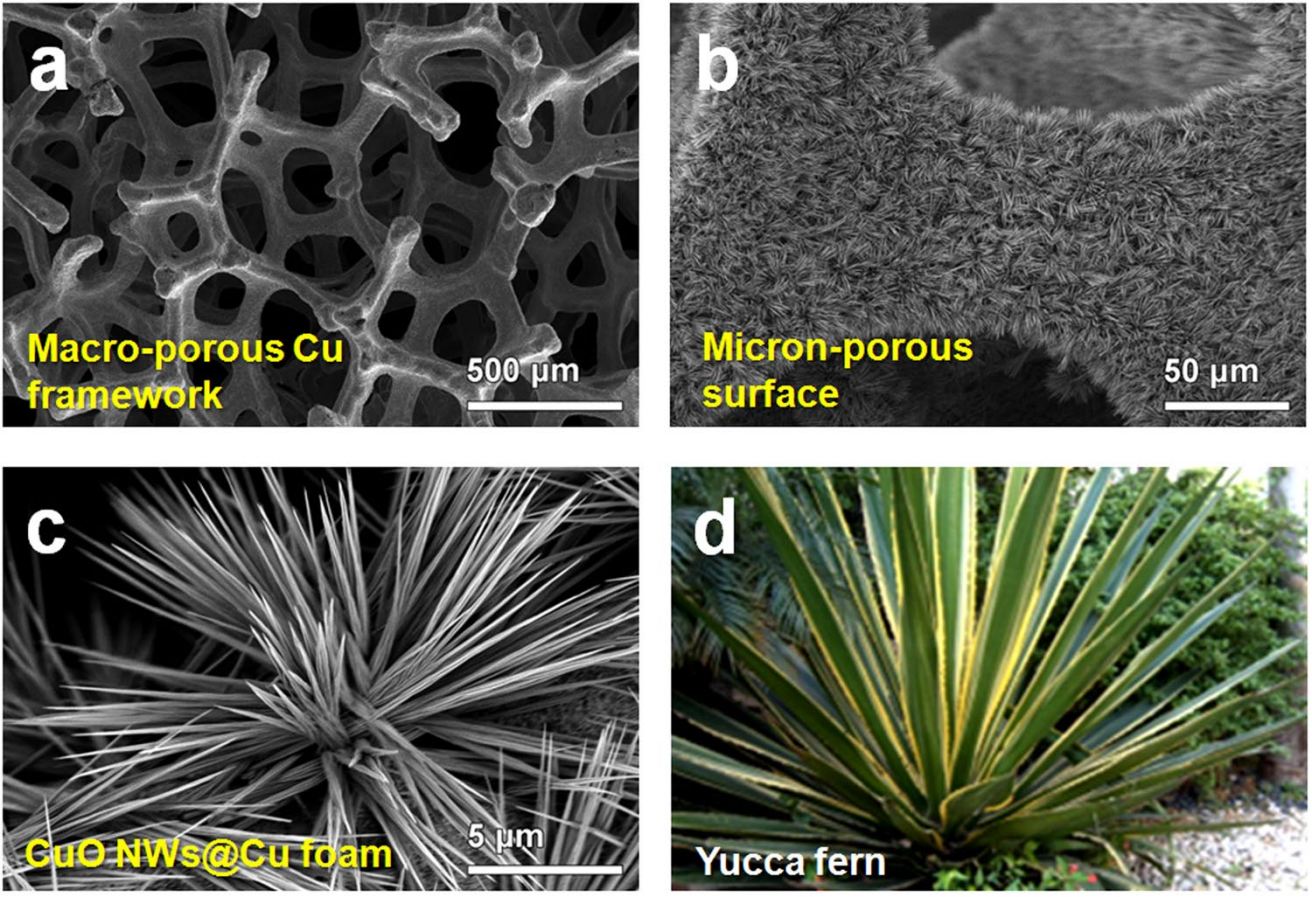
(**a**) SEM image of bare Cu foam, (**b,c**) Low- and high-magnification SEM images of CuO NWs grown on Cu foam, (**d**) Digital photograph of Yucca fern.

**Figure 3:**
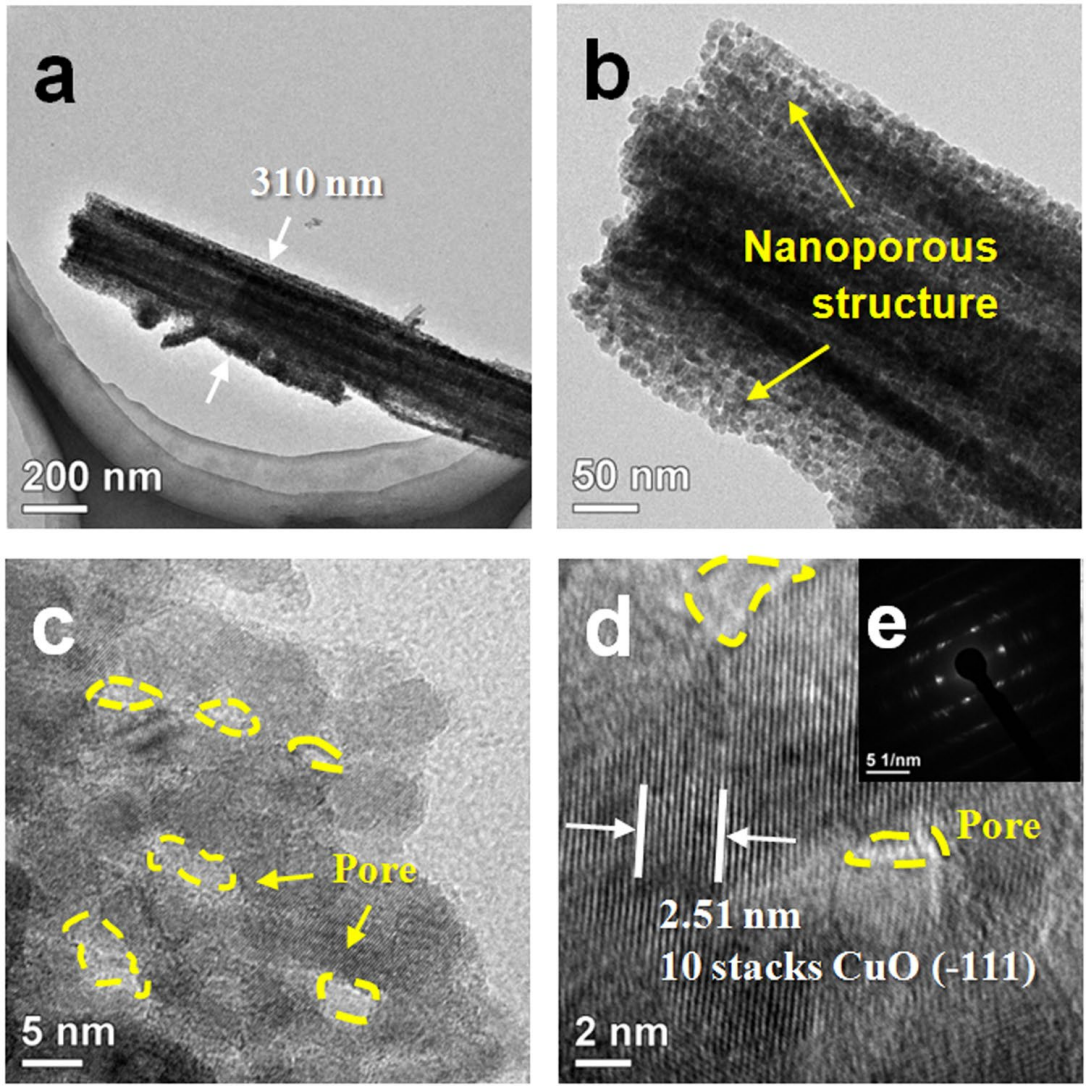
TEM images of porous CuO NWs at different magnifications (**a–d**), (**e**) SAED pattern of the porous CuO NWs.

The surface morphology of the bare Cu foam and the as-obtained CuO NWs@Cu foam was characterized by SEM and TEM. As the substrate, Cu foam was first characterized by SEM as shown in Fig. 2a. A smooth surface and 3D interconnected network with 50–80 micrometers ligament size and 200–500 micrometers pore size has been confirmed. After the anodization and calcination, uniform CuO NWs with a high density have been fabricated as displayed in Fig. 2b. A higher magnification image of CuO in Fig. 2c shows that tens of CuO NWs construct a yucca fern shaped cluster, which induce a micron-sized porous superficial structure on Cu foam. The diameter of a single CuO wire is about 310 nm as shown in Fig. 3a. A further testing of Fig. 3b and c reveals a porous feature of the CuO NWs. The pore size ranges from 2 to 10 nm. Figure 3d presents a high resolution TEM image of a CuO nanowire. The measured lattice spacing is 0.251 nm, which corresponds to the (−111) lattice plane of monoclinic CuO^22^. The SAED pattern in Fig. 3e demonstrates that the CuO NWs are highly crystalline. Finally, a 3D hierarchical porous CuO structure, containing Cu foam framework in a few hundred micrometer scale, CuO wire cluster in a micron-size scale, as well as CuO NWs with mesopores and micropores, has been achieved.

The above 3D yucca fern shaped CuO structure is deliberately designed for remitting volume expansion during lithiation/delithiation process, but it is not easy to obtain. Although anodization is not a new technology to fabricate nanowires^21^, it is firstly reported in this study that such method can be used in the preparation of radial CuO nanowires in strict technological conditions. Figure S2 presents superficial morphologies of CuO@Cu foam anodes in different processing variables. It was found that electrolyte concentration and anodization time only change the size of the obtained nanowires, while temperature and current density are more likely to control the morphologies of the anodized products. By controlling a moderate growth and leaving proper room among adjacent nanowires, yucca fern shaped CuO structure with plentiful interspaces was obtained successfully.

**Figure 4:**
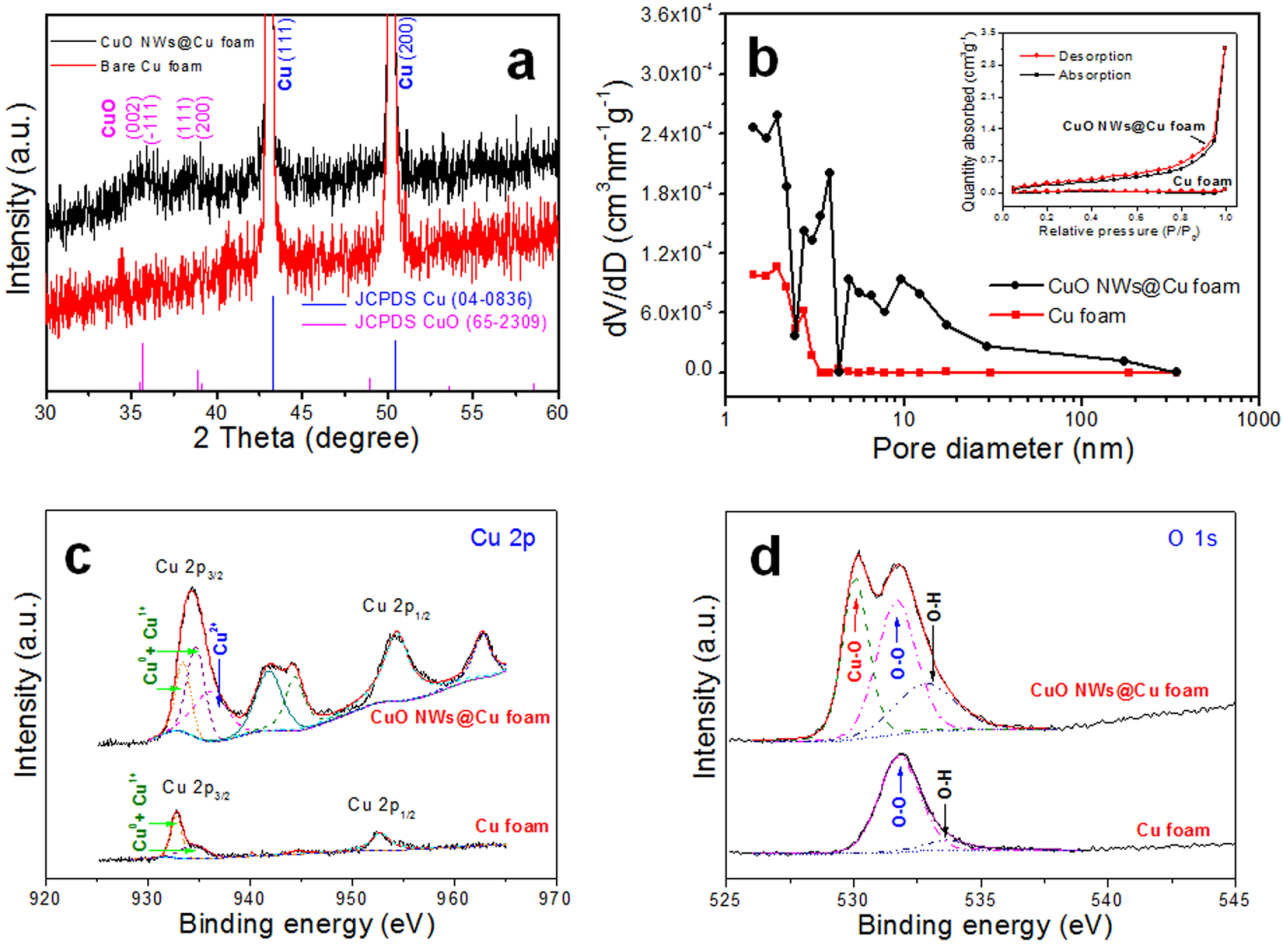
(**a**) XRD patterns of bare Cu foam and CuO NWs@Cu foam, (**b**) N_2_ adsorption desorption isotherms and the corresponding BJH pore-size distribution curves (insets) of CuO NWs@Cu foam, (**c**) Cu 2p and (**d**) O 1s XPS spectra of bare Cu foam and CuO NWs@Cu foam.

The crystal structure of CuO was further investigated by XRD as shown in Fig. 4a. The main diffraction peaks located at 43.3° and 50.4° can be indexed to the (111) and (200) planes of Cu phase (JCPDS file No. 04-0836). After the anoidzation and calcination, the diffraction peaks positioned at 35.5°, 35.6°, 38.8° and 39.1° can be assigned to the (002), (−111), (111) and (200) planes of CuO phase (JCPDS file No. 65–2309), respectively. The EDS results also presented that only Cu and O elements exist, as shown in Fig. S3. These results indicate that the CuO, without any other impurities, has been successfully obtained. The porous feature of the 3D hierarchical CuO NWs was studied by N_2_ adsorption-desorption measurements as displayed in Fig. 4b. The pore size ranges from 2 to 20 nm in the CuO NWs@Cu foam composites. Meanwhile, there are no obvious nanopores on the Cu foam substrate. The isotherm of the composites also exhibits a broad hysteresis as displayed inset of Fig. 4b, which is also the characteristic of porous structures^23^. The nanopores might greatly improve the electrolyte permeation and provide space for volume expansions during battery cycling. To further gain the surface information on the electronic state of the CuO NWs, the XPS analysis was performed as shown in Fig. 4c and d. For the bare Cu foam, the Cu 2p spectrum presents two sharp peaks located at 932.8 and 952.7 eV which are assigned to Cu 2p_3/2_ and Cu 2p_1/2_, respectively. On the other hand, the Cu 2p_3/2_ and Cu 2p_1/2_ peaks of Cu 2p electrons for the CuO NWs@Cu foam anode shift to the higher binding energies of 934.2 and 954.3 eV, respectively. Strong Cu^2+^ satellite peaks located at 941.8, 944.3 and 962.8 eV are observed in the Cu 2p spectrum of the CuO NWs@Cu foam electrode, confirming the presence of CuO on the anode surface^15^. The core-level spectrum for O 1s is displayed in Fig. 4d. The main peak of O 1s observed at 529.9 eV corresponds to the binding energy value of Cu–O, which is a further evidence of the presence of CuO on the surface of Cu foam. Other two peaks at 531.6 and 533 eV observed with higher binding energy values are associated to OH groups and to water absorbed onto the surface of the CuO NWs, respectively^24^. For the bare Cu foam, only peaks at 531.7 and 533.5 eV, which may originate from oxygen and moisture in air adsorbed on the Cu foam surface^25^, have been observed. Thus, possible existence of CuO on the Cu foam surface can be ruled out.

**Figure 5:**
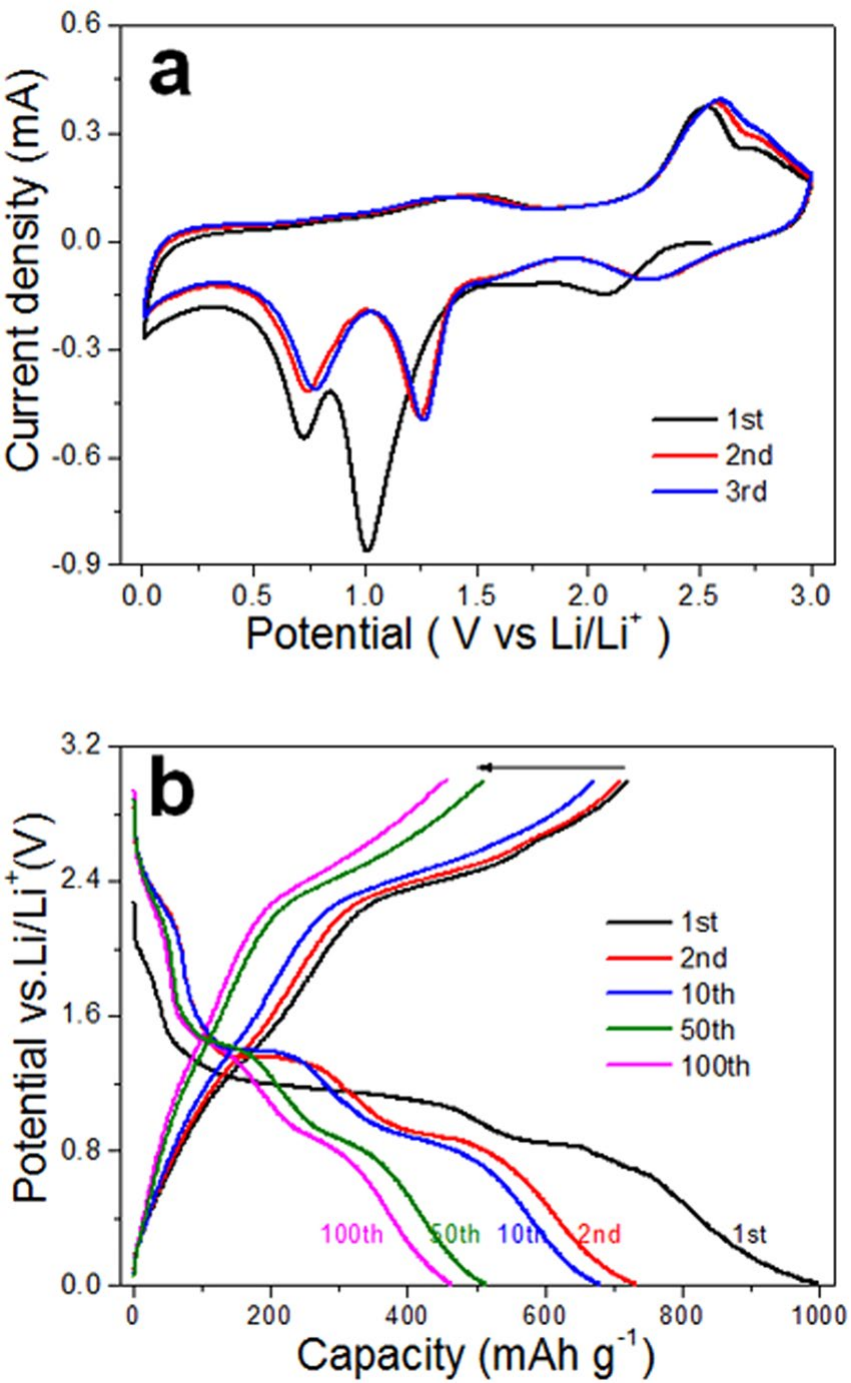
(**a**) CV curves of CuO NWs@Cu foam at a scan rate of 0.1mVs^−1^ in the potential range from 0.01 to 3.0 V vs Li/Li^+^, (**b**) Charge–discharge curves of CuO NWs@Cu foam at a current density of 100 mAg^−1^.

The Li ions storage properties of the Cu NWs@Cu foam are carefully evaluated in coin-type cells. The cyclic voltammetry (CV) curves conducted between 0.01 and 3.0 V at a scan rate of 0.1 mV/s are displayed in Fig. 5a. It clearly exhibits that three electrochemical reduction steps accompany the lithiation process. During the first cathodic sweep, a broad but weak peak appears at about 2.1 V which belongs to the initial formation of Li_*x*_ CuO^26^. Then, two succession reduction peaks are observed at 1.0 V and 0.7 V, which are associated with the further conversion reaction with deep lithiation. A solid electrolyte interphase (SEI) layer formed during the initial discharge process which results in the broaden reduction peaks. During the de-lithiation process, two main peaks at 1.5 V and 2.5 V, and a shoulder peak at 2.7 V can be observed, which corresponds to the oxidation of Cu to Cu_2_O and CuO. In the subsequent CV cycles, the reduction peaks show a positive shift and demonstrate super reproducibility, indicating the improved kinetics and good reversibility.

**Figure 6:**
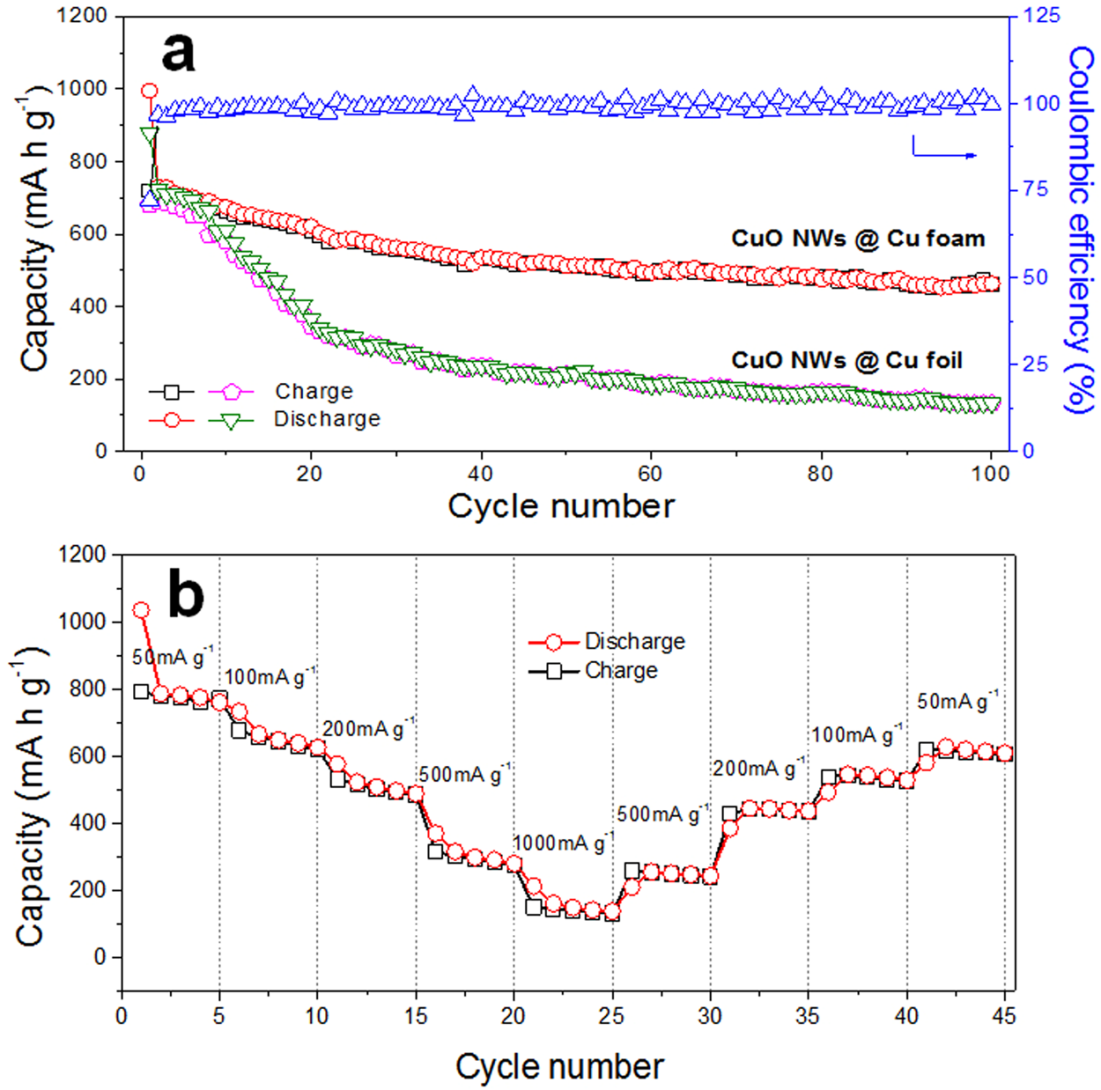
(**a**) Cycling performance comparison of CuO NWs@Cu foam and CuO NWs@Cu foil at 100 mAg^−1^, (**b**) Rate capability of CuO NWs@Cu foam electrode at different current densities from 50 to 1000 mAg^−1^.

The representative galvanostatic discharge/charge curves of the as-fabricated CuO NWs@Cu foam anode are shown in Fig. 5b. There are three plateaus during the initial discharge process, which mainly involve the continuous formation of LiCu_*x*_O and Cu_2_O phase, as well as the completely conversion to Li_2_O and Cu, respectively^17^. It is also consistent with the results of CV curves. The first discharge and charge capacities are 996.2 and 719.3 mAh/g, which gives the initial Coulombic efficiency of 72.2%. The irreversible discharge capacity is associated with the formation of SEI layer. Two charging plateaus at 2.3 V and 2.6 V are observed during the first charging process. In the subsequent cycles, it shows well cycling reversibility and almost similar discharge/charge capacities at the same cycle. The calculated Coulombic efficiency remains 99.57% last to 100 cycles (Fig. 6a), which indicates the super reversibility. As shown in Fig. 6a, the cycle performance of CuO NWs@Cu foam and CuO NWs@Cu foil substrates were carefully compared. The CuO NWs@Cu foam electrode displayed good cyclic stability and a discharge capacity of 461.5 mAh/g even at the 100th cycle. In contrast, the CuO NWs@Cu foil electrode gave similar capacities during the initial 10 cycles. However, there is a gradually capacity fading in the following cycles. The capacity retention is only 19.5% at the 100th cycle for the CuO NWs@Cu foil. This comparison results indicate that the 3D structure can be better accommodation for the electrode volume expansions during discharge/charge cycles. The rate capability of the CuO NWs@Cu foam was further investigated at varied current densities (Fig. 6b). The first charge capacities are 792.6, 678.7, 532.2, 315.5 and 150.6 mAh/g at the current densities of 50, 100, 200, 500 and 1000 mA/g, respectively. There are two reasons for the excellent rate performance: firstly, the 3D structure features improve the electrolyte permeation and accommodate the volume expansion; secondly, the Cu foam substrate and the Cu components at the final discharge state obviously enhance the conductivity of the electrode composite. When the current density returned back to 50 mA/g, 77.9% of the initial charge capacity was regained. The slight performance fading was due to the formation of stable SEI interface layers. In order to clearly evaluate the performance of CuO NWs@Cu foam electrode, a detailed comparison between the present electrode and the reported CuO electrodes based on different synthesis methods and structures is shown in Table 1^15,17,25,27–32^. The superiority of the freestanding hierarchical porous structure of the present electrode, which guaranteed excellent electrochemical properties, can be seen from the table.

**Figure 7:**
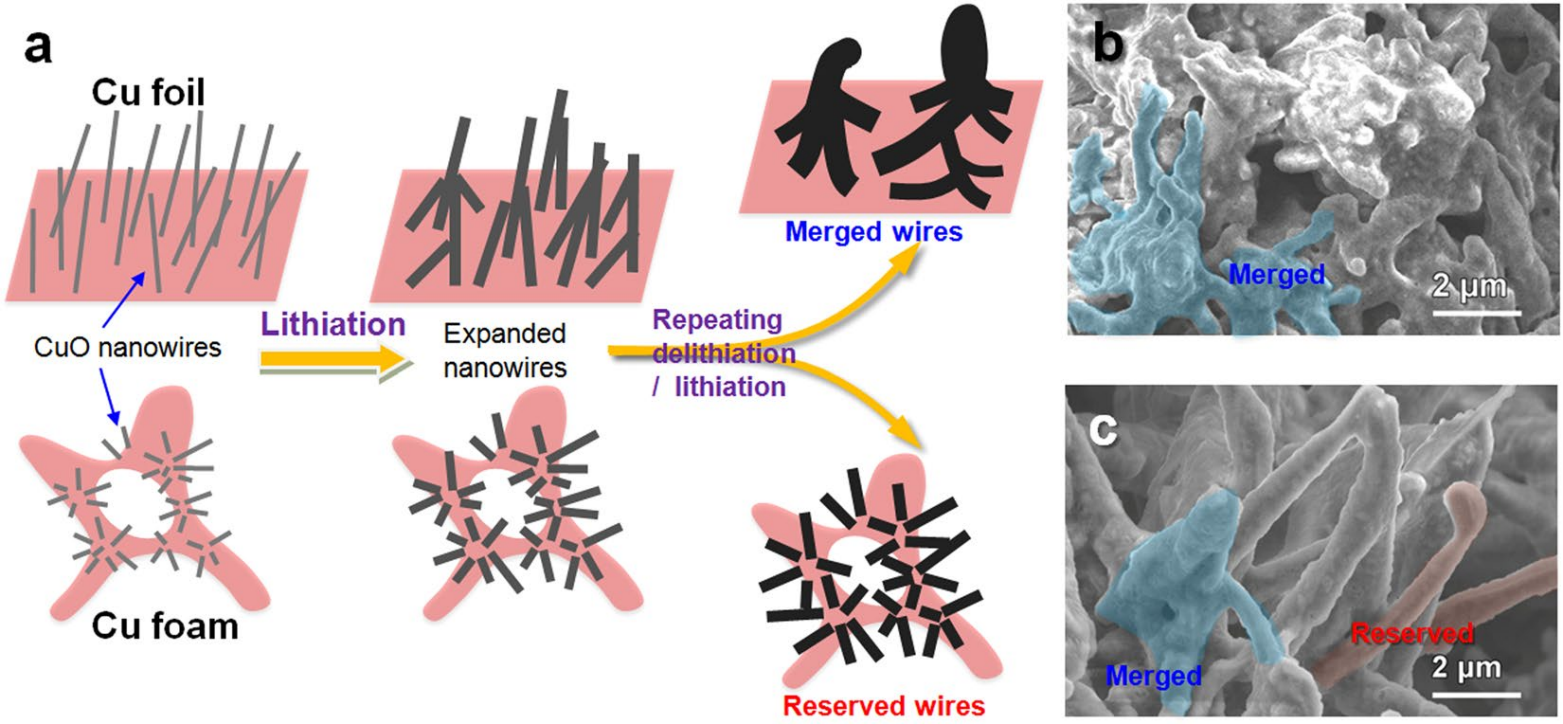
(**a**) Schematic comparison of the morphological and volumetric changes of CuO NWs@Cu foil and CuO NWs@Cu foam, (**b**) SEM image of CuO NWs@Cu foil after 110 charge-discharge cycles, (**c**) SEM image of CuO NWs@Cu foam after 110 charge-discharge cycles.

To further understand the performance improvement from the Cu foil to the 3D Cu foam substrates, the electrode morphologies after discharge/charge 110 cycles were investigated by SEM as depicted in Fig. 7. The cycled samples were first disassembled in Ar-filled glove box and washed by electrolyte-free solvent. The surface of the CuO nanowires were coated by a solid layer and the surface became rough for both of the samples. This rough layer is mainly originated from the decomposition of the electrolyte during the initial discharge cycles. Obviously, some of the thick wires intertwined and merged together to form squid-shaped lumps, which resulted in the performance fading. It is much severe with a Cu foil substrate because of the inter-cross original architectural feature of the CuO NWs as shown in Fig. S4. It is nice to see that the 3D foam architecture and the yucca fern shaped CuO cluster alleviated this fading tendency and the morphology maintained better, although the nanowire seems to be expanded to some extents. The expansion is due to the unavoidable volume change of transition metal oxides during lithiation/delithiation process as well as the protective effects of stable SEI films. The nanowire grows up a little at every repeated cycle so that it cannot recover to its initial state. The accumulative effect of 110 repeated cycles finally leads to this clear expansion. A further observation on the macro-photograph of CuO NWs@Cu foam anode before and after 110 cycles is shown in Fig. S5. It revealed that there is a good binding force between CuO active material and the Cu foam substrate. Barely cracking-off problem is observed for the CuO NWs@Cu foam anode. These results are consistent with the electrochemical performance.

The internal resistance evolutions of the two samples with different Cu current collectors were also analyzed by electrochemical impedance spectrum (EIS). As shown in Fig. S6, the Nyquist plots are composed of a semicircle followed slope lines, which correspond to the charge-transfer resistance and the Warburg behaviors. It can be seen that two kinds of fresh electrodes exhibit almost the similar resistance (Fig. S6a). After 110 repeated cycles (Fig. S6b), both the resistance reduces to a certain extent as compared to the fresh ones. It can be found obviously that after cycles, the resistance of the electrode with Cu foam current collector is much lower than that of electrode with Cu foil current collector. The 3D Cu foam substrate dramatically reduced the resistance, which indicates the better electrochemical kinetics of the Cu NWs@Cu foam than that of CuO NWs@Cu foil.

## Discussion

### Formation mechanism of Yucca fern shaped CuO

The structure of CuO was mainly inherited from Yucca fern structural Cu(OH)_2_ framework. So, the study on the formation mechanism of the Yucca fern shaped CuO is indeed an investigation on how Cu(OH)_2_ framework created.

Every Yucca fern structural Cu(OH)_2_ cluster is comprised by tens of nanowires. The microscopic formation mechanism of the nanowire is analyzed as follows. The nucleus shape of monoclinic Cu(OH)_2_ crystal is usually a rod-like structure containing a tip. Under a moderate anodizing condition, most of Cu ions were transported to tips, resulting in a quick growth of crystals in vertical direction. Only a small part of crystals grow in diameter direction at the same time, so that the growth rate of crystals in vertical direction obviously higher than that in diameter direction. As a result, it is easy to form a wire-like structure under current conditions. If the experimental conditions are changed, the growth rate of crystals in different growth directions may change too. On this occasion, Cu(OH)_2_ crystals with possible shape of nanoplate and nanosphere instead of nanowire may be generated.

The macroscopic formation mechanism of Yucca fern structural Cu(OH)_2_ is analyzed as follows. Under suitable anodizing conditions, partial Cu atoms at the Cu foam surface were oxidized to Cu^2+^ ions and immediately reacted with OH^−^ from the alkaline solution to form Cu(OH)_2_ crystals and deposited on the surface of Cu foam. With the increase of the anodizing time, Cu(OH)_2_ kept growing in the solution and formed short nanorods. Cu(OH)_2_ nanowires were then generated by further growing of these nanorods. Controlling the number of nucleation sites through adjusting anodizing parameters, Cu(OH)_2_ nanowires were designed to nucleate and grow only at limited sites. Generally, a nucleation site develops into more than one nanowire. Similar to the human hair follicles, one nucleation site is easy to grow into a cluster of radial nanowires, which is named Yucca fern structural nanowires in this study. It needs to be emphasized that the anodizing parameters have an important influence on the formation of Yucca fern structural nanowires. Other morphologies of Cu(OH)_2_ such as nanoplates, nanowire arrays instead of Yucca fern shape may be prepared by adopting improper experimental conditions.

### Importance of Yucca fern structure

The hierarchical CuO NWs with Yucca fern structure contains many advantages. Firstly, the 3D structure features can improve the electrolyte permeation and accommodate the volume expansion, which guarantee a good cycling stability. Secondly, the Cu foam substrate and the Cu components at the final discharge state greatly enhance the conductivity of the electrode composite, resulting in a good rate property. Based on the results of this work, the relatively stable Yucca fern structure before and after cycling is the key to maintain capacity stability, indicating the significance of this structure in restraining the capacity fading. In addition, in terms of preparation technology, the anodizing is a simple and rapid process to prepare low-cost anodes, which may facilitate the assembly line production.

If the structure cannot be remained, there must be a clear capacity fading. After 110 repeated cycles, a small part of nanowires had merged together and transformed into squid shape. So, we cannot rule out the possibility of a capacity fading even by utilizing the as-obtained Yucca fern structure during next hundreds of cycles. Anyway, the material has shown excellent performances compared to the existing reports (Table 1). In future work, graphene and other carbon materials were planned to coat on the surface of Yucca fern structural CuO for improving the long-term stability in both the structure and capacity.

**Table 1:** Comparison of capacities of the present work with previous CuO electrodes reported in the literatures.

CuO materials	Synthesis method	Current density (mA g^−1^)	Cycle number	Capacity (mA g^−1^)	Ref.
Leaf-like	Hydrothermal method	67	55	421	^25^
Flower-like	Hydrothermal method	67	50	392.4	^17^
Octahedral crystals	Reduction and calcination	134	50	440	^26^
Nanoplates	Hydrothermal method	335	50	281.6	^15^
Dandelion-like	Hydrothermal and calcination	67	50	400	^27^
Hollow nanospheres	Cu_2_ O oxidation	150	50	91	^28^
Spherical	Pyrolysis of MOF	100	40	500	^29^
Core-shell nanowires	Hydrothermal and oxidation	100	50	345	^30^
Bowknot-like	Solution route and calcination	100	30	470	^31^
CuO NWs@Cu foam	Anodization and calcination	100	50	510.1	This work
CuO NWs@Cu foam	Anodization and calcination	100	100	461.5	This work

## Conclusions

In summary, a facile and scalable approach for the fabrication of freestanding 3D yucca fern shaped CuO nanowires with hierarchical porous structure has been introduced. The possible Cu source and substrates have been investigated. The obtained CuO NWs@Cu foam electrode demonstrated superior electrochemical properties, including stable cycle performance (461.5 mAh/g after 100 cycles) and high rate capability (150.6 mAh/g at current density of 1000 mA/g). The outstanding electrochemical could be attributed to hierarchical porous structured nanowires combined a 3D foam substrate. This unique structure feature guaranteed this Cu NWs@Cu foam a promising anode with a high performance for lithium ion batteries. In addition, the simple fabrication process involving mature anodization technology boosts prospects for scalable production.

## Methods

### Materials preparation

The bare Cu foam with thickness of 0.8 mm was purchased from Kunshan Jiayisheng Electronics Co. Ltd., China. The anodization was carried out by DC Power Supply (TPR-12010D). Cu foam (2.0 cm × 4.0 cm) was successively washed with acetone, ethanol, 0.5 M HCl, and deionized water for 10 min under ultrasonic condition to remove organic contaminants and an oxide layer on surface. And then it was dried using a stream of a compressed air and before using as the working electrode. Another piece of Cu foam with a same size was selected as the counter electrode. The electrolyte was 1.0 M KOH solution, which was deaerated by bubbling with dry argon for at least 20 min before experimentation. The Cu foam was electrochemically anodized at a constant current density of 10 mA cm^−2^ with a typical reaction time of 10 min to form Cu(OH)_2_ NWs. A thermostat was used to control cell temperature at 20 ± 1 °C. After anodization, samples were rinsed twice with deionized water and then carried out a calcination treatment. The calcination temperature was maintained at 185 ± 2 °C for 1.5 h to converted Cu(OH)_2_ to CuO. Finally, a binder free CuO NWs@Cu foam anode was obtained. The total weight of an anode is about 55 mg, in which the weight of active materials is about 6 mg. A CuO NWs@Cu foil anode was also obtained through a same process for comparison. All the chemicals were of analytical grade and used as purchased without further purification.

### Materials characterization

The morphologies of the as-prepared CuO NWs and Cu substrates were observed by scanning electron microscopy (SEM, Nova nanoSEM 450, FEI) equipped with an X-ray energy dispersive spectroscope (EDS) and transmission electron microscopy (JEM-2100F, JEOL). X-ray diffraction (XRD) patterns were obtained using an X-ray diffractometer (D/Max-2500, Rigaku) with Cu K *α* radiation to confirm crystalline structures. X-ray photoelectron spectroscopy (XPS, Thermo Fisher Scientific) was employed to determine the chemical composition and valence state of the anodized products. Nitrogen adsorption/desorption isotherms were measured at 77.2 K with a physisorption analyzer (Micromeritics ASAP 2020). The specific surface area was calculated using the Brunauer–Emmett–Teller (BET) method. The pore size distribution was obtained by the Barrett–Joyner–Halenda (BJH) method.

### Electrochemical testing

Electrochemical performances were examined using coin cells (CR2032) assembled in an argon-filled glove box. The cell was consisted of CuO NWs@Cu foam as a working electrode and Li foil as both counter and reference electrode. The electrodes were separated by a Celgard 2400 separator. The electrolyte is composed of a solution of 1 M LiPF_6_ in a mixture of ethylene carbonate (EC)/dimethyl carbonate (DMC) (1:1, v/v) (Shanghai Xiaoyuan Energy Technology Ltd., China). The cyclic voltammetry (CV) test was carried out on an electrochemical workstation (CHI660B, China) from 0.01 to 3V. Electrochemical impedance spectroscopy (EIS) measurements were performed on electrochemical workstation (Princeton Applied Research, PARSTAT 2273), and the frequency ranged from 10 mHz to 100 kHz with an applied AC signal amplitude of 5 mV. The charge-discharge test was measured by using a battery testing system (LAND CT2001A, China) in the voltage range of 0.01–3.0 V (vs. Li^+^/Li) under different current densities.

## Electronic supplementary material

Electronic supplementary information
