# Supplementary material for: Yucca fern shaped CuO nanowires on Cu foam for remitting capacity fading of Li-ion battery anodes
Source: Sci Rep. 2018 Apr 25;8:6530. doi: 10.1038/s41598-018-24963-2 (PMC5916934; doi:10.1038/s41598-018-24963-2)
Supplement: Supplementary file 1 — Electronic supplementary information [file 41598_2018_24963_MOESM1_ESM.doc]

**Electronic supplementary information (ESI)**

**Yucca fern shaped CuO nanowires on Cu foam for remitting capacity fading of Li-ion battery anodes**

Zhifeng Wang ^1,2^, Yanshan Zhang ^1^, Hanqing Xiong ^3^, Chunling Qin ^1,*^, Weimin Zhao ^1^,

Xizheng Liu ^2,4,*^

*^1^ Key Laboratory for New Type of Functional Materials in Hebei Province, School of Materials Science and Engineering, Hebei University of Technology, Tianjin 300130, China, Email: clqin@hebut.edu.cn*

*^2^ Tianjin Key Laboratory of Advanced Functional Porous Materials, Institute for New Energy Materials & Low-Carbon Technology, School of Materials Science and Engineering, Tianjin University of Technology, Tianjin 300384, China, Email: xzliu@tjut.edu.cn*

*^3^ School of Materials Science and Engineering, Central South University, Changsha 410083, China*

*^4^ Key Laboratory of Advanced Energy Materials Chemistry (Ministry of Education), Nankai University, Tianjin 300071, China*

* Corresponding author:

**Chunling Qin**, Prof., Email: clqin@hebut.edu.cn, ORCID: 0000-0003-3525-7802.

**Xizheng Liu**, A/Prof. Email: xzliu@tjut.edu.cn, ORCID: 0000-0001-5641-3849.

**ESI Figure Captions**

Fig. S1 Macrophotograph of sample changes during preparation process.

Fig. S2 Superficial morphologies of CuO@Cu foam anodes in different processing variables:

(a-c) temperature, (d-f) electrolyte concentration, (g-i) time, (j-l) current density.

Fig. S3 EDS analysis of the as-obtained CuO NWs@Cu foam anode.

Fig. S4 SEM image of CuO NWs@Cu foil anode.

Fig. S5 Macrophotograph of CuO NWs@Cu foam anode before and after 110 cycles.

Fig. S6 Nyquist plots of CuO NWs@Cu foam and CuO NWs@Cu foil: (a) fresh, (b) after 110 cycles.


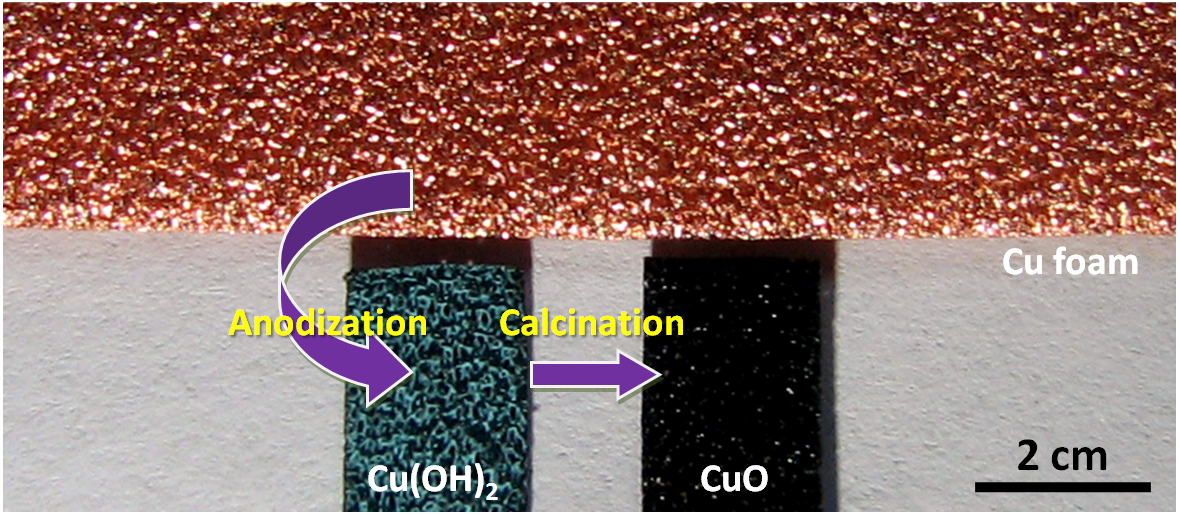


Fig. S1


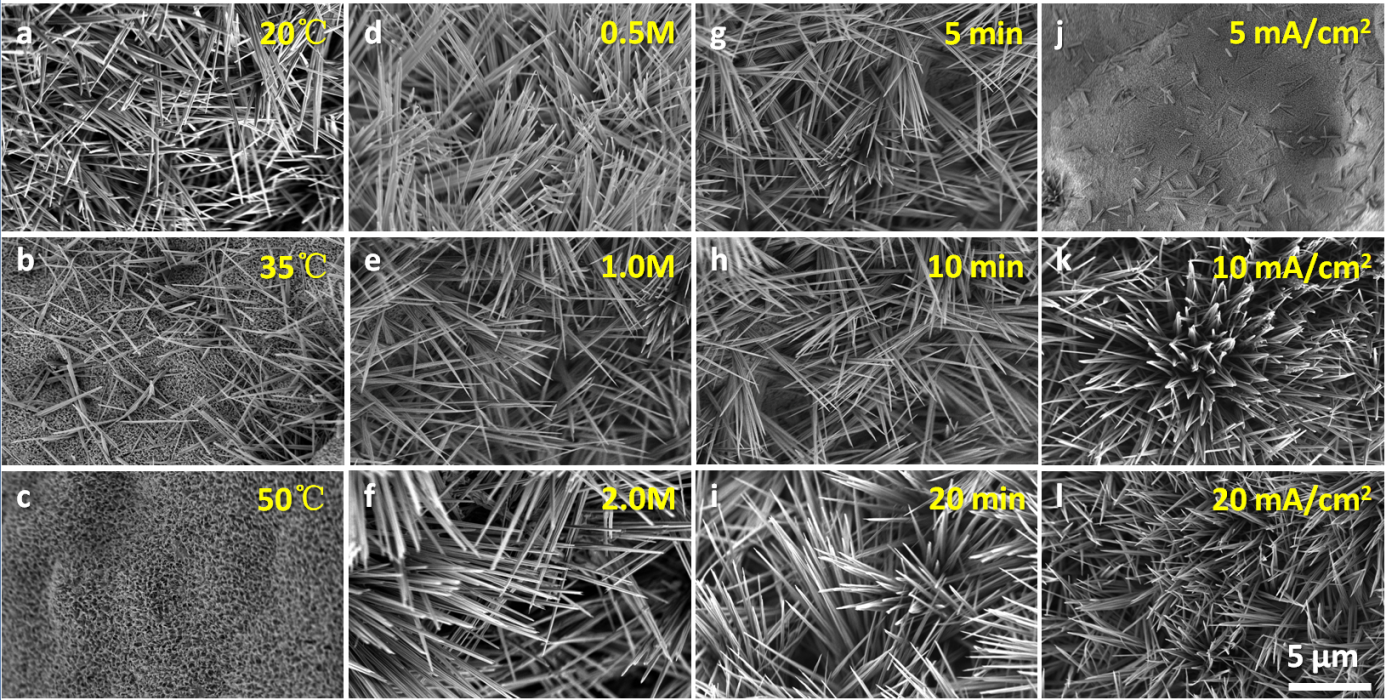


**Radial-like**

Fig. S2


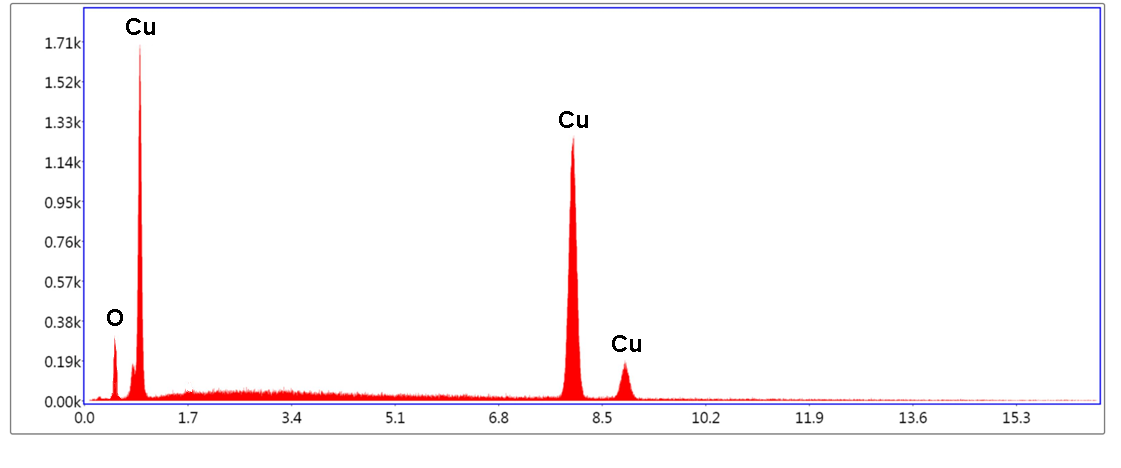


Fig. S3


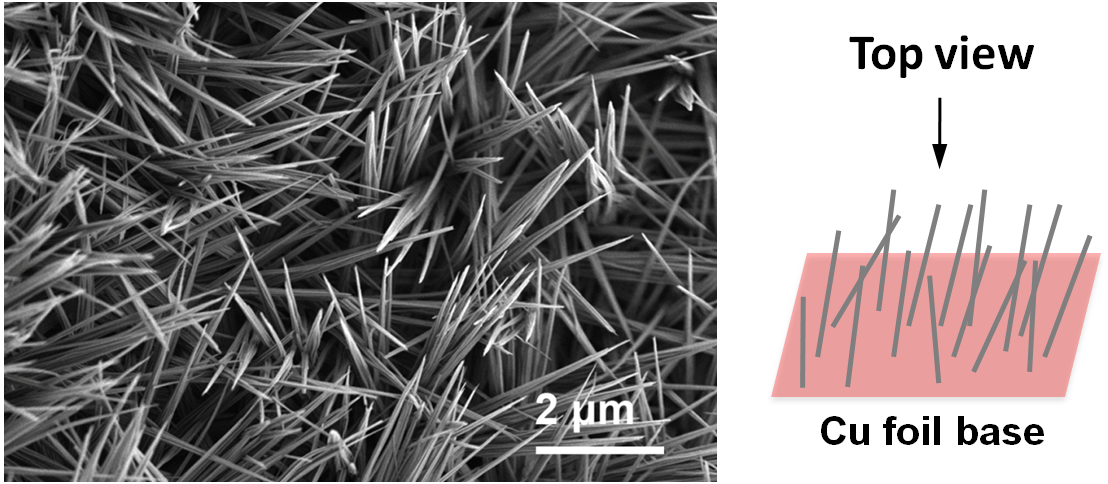


Fig. S4


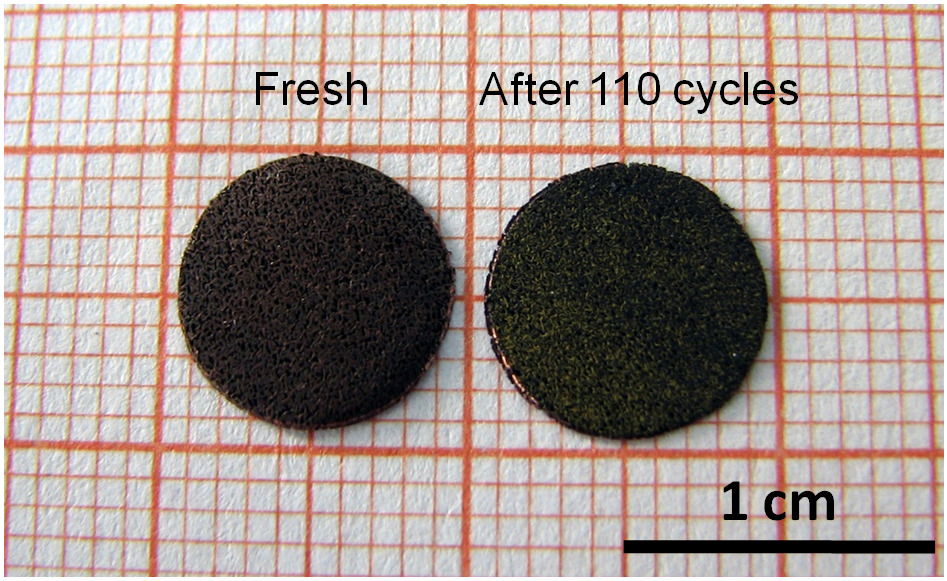


Fig. S5


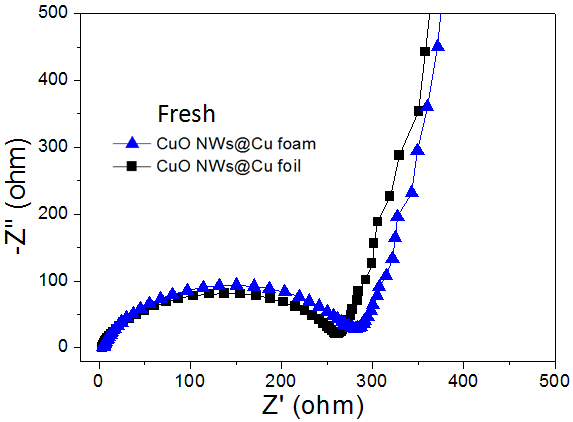


**a**


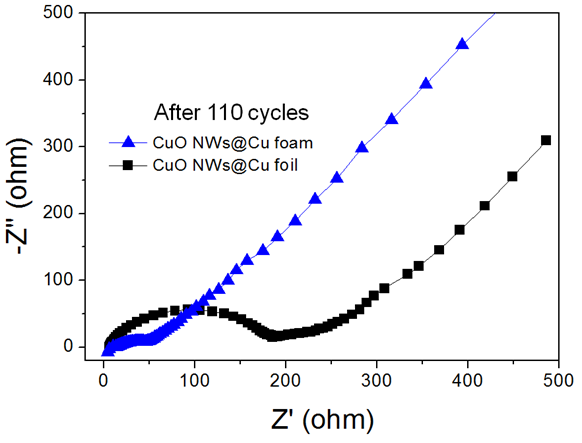


**b**

Fig. S6
